# Awareness and willingness to use HIV oral pre-exposure prophylaxis among people who inject drugs in Dar es Salaam, Tanzania: A cross-sectional survey

**DOI:** 10.1371/journal.pgph.0000776

**Published:** 2022-11-22

**Authors:** Masunga K. Iseselo, Edith A. M. Tarimo, Eric Sandstrom, Asli Kulane

**Affiliations:** 1 Department of Clinical Nursing, Muhimbili University of Health and Allied Sciences, Dar es Salaam, Tanzania; 2 Department of Nursing Management, Muhimbili University of Health and Allied Sciences, Dar es Salaam, Tanzania; 3 Department of Global Public Health, Equity and Health Policy Research Group, Karolinska Institutet, Stockholm, Sweden; 4 Department of Clinical Science and Education, Södersjukhuset, Karolinska Institutet, Stockholm, Sweden; Chinese Academy of Medical Sciences and Peking Union Medical College, CHINA

## Abstract

People who inject drugs (PWID) are at increased risk of HIV infection. Pre-exposure prophylaxis (PrEP) could help in HIV prevention among PWIDs. However, little is known about PrEP use among PWIDs in low and middle-income countries. This study reports the awareness of and willingness to use PrEP and the associated factors among PWID in Tanzania. A cross-sectional survey was conducted using respondent-driven sampling (RDS) to recruit PWIDs in Dar es Salaam, Tanzania. Data were collected using an interviewer-administered questionnaire. Chi-square statistical test was used during data analysis. The P-value of < 0.05 was used to ascertain the statistically significant relationship. IBM SPSS Statistics 25.0 was used to analyze the data. The analysis consisted of 260 PWIDs. The mean age of the respondents was 39.0 years with a standard deviation (SD) of ±7.5. Most of the respondents were male (n = 232, 89.2%) with primary education (n = 176, 67.7%). Despite the low awareness of PrEP (n = 42, 165.28%) in the study sample, the majority (n = 239, 91.9%) were willing to use PrEP. Both awareness of and willingness to use PrEP were associated with gender (*p* = .002 and *p* = < .001), awareness of HIV prevention programs(*p* = < .001 and *p* = .006), selling sex (*p* = .010 and *p* = .021), and frequency of condomless sexual intercourse (*p* = .029 and *p* = .025) respectively. In multivariable logistic regression, only gender*(p* = 0.046) was related to awareness of PrEP while awareness of HIV prevention programs (*p* = 0.009), the risk level of HIV infection(*p* = < .001), number of sexual partners(*p* = 0.046), and frequency of condomless sex(*p* = 0.032) were associated with willingness to use PrEP. Other factors were not statistically significant. Despite low awareness, PWIDs are highly willing to use PrEP. Future research should assess the acceptability of injectable PrEP for PWID, as their acquaintance with injection may make the formulation more practical.

## Introduction

Human Immunodeficiency Virus (HIV) continues to be a major global health concern despite intense efforts in global and local initiatives to address the pandemic. Approximately 37.7 million individuals were living with HIV and 1.5 million were newly infected globally in 2020 [1]. Sub-saharan Africa accounts for almost 60% of the global new HIV infections [1]. Tanzania’s HIV epidemic affects all sections of society [2], but the prevalence is high among people who inject drugs(PWIDs) [3–5]. In 2014, it was estimated that 35% of PWIDs were living with HIV [6]. There is a gender difference in HIV distribution among PWIDs. HIV prevalence among women who inject drugs is thought to be twice that of their male counterparts [7]. The reasons for this are not fully known although possible factors include women who inject drugs being involved in sex work or being last in line when syringes are shared [8]. This increases their chance of HIV getting infection compared to their male counterpart.

Despite antiretroviral therapy (ART) having reduced AIDS-related deaths in all risk groups [9–12], access to treatment is not universal, and the predictions of curative treatments and an effective vaccine are still unknown. It has been proposed that prevention and awareness programs may prove to be more of a practical approach [13]. Traditional methods such as condom use, reducing sexual risk behaviours, and using safe needles have been used for decades to prevent HIV infection but these have not been adequate to prevent the infection [14]. Biomedical prevention is an additional approach, with one of the most effective recent methods being oral pre-exposure prophylaxis (PrEP) [15–17]. PrEP is an antiretroviral, given to HIV-negative people at high risk of HIV infection that offers a pharmacological barrier to infection. When taken regularly, PrEP is highly effective [18], with little or no breakthrough infection observed in adherent individuals [19–21].

Pre-exposure prophylaxis has been shown to decrease the risk of HIV infection among at-risk populations, including PWID [22,23]. Despite PrEP eligibility, systematic reviews and meta-analyses among key populations reported that PWIDs have the lowest PrEP use compared to other key populations such as men who have sex with men (MSM), and transgender women [24]. Currently, there has been one efficacy trial evaluating the efficacy of PrEP among PWIDs, the Bangkok Tenofovir Study (BTS). The results from BTS showed a significant reduction in HIV incidence in PWIDs from methadone clinics taking daily PrEP compared to placebo [22]. The reduction rate increased considerably with the highest amounts of medication adherence [25] suggesting that the perceived risk of HIV infection among PWIDs may have a significant contribution to their decision to initiate PrEP [26]. Although PrEP has been demonstrated to be effective in PWIDs, awareness, and willingness to use it among this population are still low. The available evidence suggests that limited knowledge and misperceptions about the risk of acquiring HIV act as barriers for eligible PWID to receive recommended PrEP care [27,28]. Evidence from a cross-sectional survey of HIV-negative PWID reported that 90% would take PrEP every day suggesting that adherence may be feasible [29].

Studies in high-income countries have helped to determine adherence to PrEP [30,31], but few studies have assessed the acceptability and use of PrEP in low-income countries. The study in Kenya reported that the acceptability of PrEP among MSM and female sex workers was high with multiple challenges in adherence and social impacts [32]. In 2018 Tanzania began to implement PrEP for HIV-negative key populations to protect themselves from infection. The following year(2019), the government announced plans to extend this nationwide, including expanding eligibility criteria to include adolescent girls and young women [33]. Implementation of PrEP started with limited information among the target population. For example, a study among female bar workers in Dar es Salaam revealed that awareness of PrEP for HIV prevention was very low but interest in PrEP, particularly for daily pills and long-acting injectables, was high [34]. Another study in Mwanza revealed that disclosure of PrEP use to family members and friends was common among young females to secure social support and advocate PrEP uptake among the at-risk population [35]. Unfortunately, none of these studies has assessed the awareness and willingness to use PrEP for HIV prevention among PWIDs in low-income countries. This lack of research in the local context results in a knowledge gap about effective strategies for PrEP initiation among PWIDs. Therefore, this study reports on the awareness and willingness to use PrEP among PWIDs in Dar es Salaam, Tanzania.

## Materials and methods

### Design and setting

The cross-sectional survey was conducted to determine the awareness and willingness to use PrEP among PWID. This method was useful to make inferences about possible relationships between individual characteristics, risk behaviours, and willingness to use PrEP for HIV infection and yead yield relatively faster results than any other design [36]. This study was conducted in Dar es Salaam, a region with an estimated total population of about 7 million people and a growth rate of about 5% higher than that of the general population (3%) [37]. The growth of the city is construed by both natural increases and a high rate of migration. It is the leading commercial centre and economic hub in Tanzania and is expected to be a mega city by 2030 [38]. In 2018, HIV prevalence in Dar es Salaam city was 4.7% which was almost similar to that of the general population (4.6%) [39].

### Study population

People who inject drugs were screened and recruited from the community to participate in the study. Eligible respondents were those who self-reported to be HIV negative, 18 years and above, lived in Dar es Salaam in the past 6 months, and had injected drugs in the past 6 months before the commencement of the study. Screening measures for drug use included self-report and visual inspection for injection marks on the arms, feet, or legs. Some potential respondents also showed us their injection equipment and drugs voluntarily, as evidence that they were drug injectors. In this case, three potential participants were excluded from the data collection site due to a lack of visible injection marks on their bodies. Those who were drug users but not injectors were similarly excluded from the study due to differences in HIV infection profile [40]. A field person (screener) determined the eligible participants according to the criteria **(S1 File)** and agreed upon the day of the interviews.

### Sampling technique and sample size

**Fig 1:**
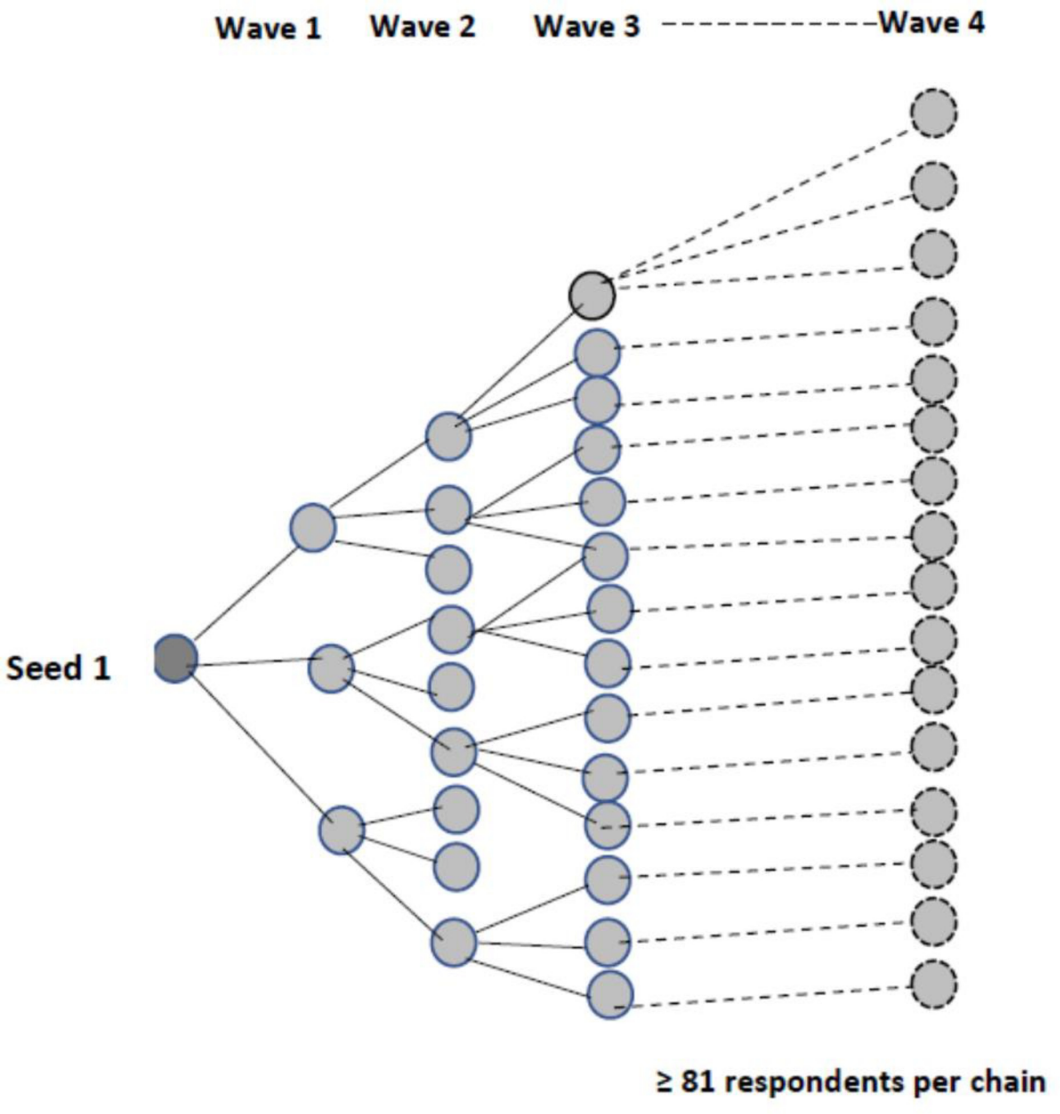
Example of the recruitment process for study participants using RDS.

We used respondent-driven sampling (RDS) to recruit the respondents. RDS is a peer chain-referral technique that is effective in accessing hidden populations and, under certain assumptions, yields unbiased population estimates when RDS weights are applied [41]. RDS was implemented in four stages. First, eligible three seeds who were well connected to other PWIDs in each location were invited to participate in the survey. Seeds were identified using a former injecting drug user (a screener). This person was conversant with the shooting galleries where PWIDs go to inject drugs in different parts of Dar es Salaam. The selection of seeds was carefully planned so that the seeds came from multiple locations to improve access to potentially hidden networks of PWIDs. Second, each seed was given a serial number of a coupon and enrolled as a participant. Third, each seed received three coupons and was asked to refer to three additional PWIDs. They would receive $1.7 as a secondary incentive for a successful referral. Finally, recruited individuals were provided with the same opportunity as seeds to recruit other PWIDs. The recruitment process was continued until the target sample size with 4 waves of recruitment was reached **(Fig 1)**.

A serial number check was used to ensure individuals did not participate in the survey more than once.

### Training of study team

All data collection team members participated in a one-day training session. The data collection team consisted of a screener (screening for eligibility criteria), two research assistants (administering the questionnaires), and a coupon manager (control of coupons). The training covered a review of the study protocol, data collection methods, tools, and question reflections. The training facilitators explained the study protocol and tools in detail during the sessions to ensure that all team members were thoroughly familiar with them. A regular daily review session with the teams was conducted during the survey period to review progress and communicate any issues encountered during the data collection.

### Measures

#### Dependent variable

For this study, the outcome variables of interest were awareness of and willingness to use PrEP among PWID.

#### Independent variables

The predictive variables were socio-demographic characteristics (i.e age, gender, marital status, education level) and high-risk behaviours of PWIDs (i.e frequency of condomless sex, number of sexual partners, self-reported HIV risk level, awareness of strategies for HIV prevention, and selling sex.

#### Data collection tool and procedure

Data collection took place between November and December 2022 at Muhimbili University of Health and Allied Sciences (MUHAS). An interviewer-administered questionnaire was used to collect the data **(S2 File)**. We used an Open Data Kit Collect (ODK, v2021.3.4). ODK Collect is an open-source Androids app that replaces paper forms used in survey-based data gathering. It supports a wide range of questions and answers and is designed to work well without internet connectivity [42]. Additionally, ODK provides no or minimal missing data outputs than paperwork [43]. The questionnaire was designed in English after an extensive review of the literature and was validated by a team of experts [44,45] which consisted of 2 researchers on PrEP, psychiatrists, and a person who injects drugs. The team of experts provided useful input that helped to modify some question items. The Kiswahili version was used during data collection after to-and-back translation procedures [46]. This is because Kiswahili is the common language used by all study respondents.

The questionnaire consisted of 44 questions on socio-demographic characteristics, drug injecting and risky sexual behaviours, awareness, and willingness to use PrEP. For awareness and willingness to use PrEP, respondents were asked whether they were aware of (i.e., had ever heard of) PrEP as a way to prevent HIV. Those who were aware of PrEP were asked where they heard about it (Response: Media, friends, health care providers, or others). A brief description of oral PrEP was provided to respondents who were not aware of it. This included the meaning, purpose, eligibility, and efficacy of PrEP in HIV prevention. Then respondents were asked whether they would be willing to take PrEP every day to reduce their risk of contracting HIV. Responses were categorized as “yes /no”. Respondents were regarded as willing if they responded “yes”. Respondents were asked related questions about the use of PrEP in terms of mild side effects, with condoms and HIV testing every three months. Subsequently, they were asked whether they would prefer PrEP taken as a pill every day or as an injection every one to two months. They were asked to give reasons for their choices. Finally, respondents were asked the total number of eligible PWIDs they had in their social network. Network size was utilized as a continuous variable. Each respondent spent an average of 30 minutes completing a questionnaire. Respondents were remunerated $3.5 for their participation.

#### Data processing and analysis

Data processing and coding immediately started after data collection. Open-ended questions were analyzed using qualitative content analysis [47] in which we read the sub-set of the responses and devised a coding frame to describe the thematic content of the responses. We then manually assigned codes to all the responses. The codes were entered into a statistical package alongside the data from the closed questions and treated as variables in quantitative analysis. To facilitate the analyses, age was transformed into categorical variables. Analysis was carried out in two stages. First, descriptive statistics were calculated for socio-demographic, behavioural risk factors, awareness of strategies for HIV prevention programs, and willingness to use PrEP. Second, bivariable analyses, using chi-squared statistics, were conducted to determine the association of awareness of and willingness to use PrEP with predictor variables (i.e socio-demographic, behavioural risk factors, awareness of strategies for HIV prevention programs). The P-value of < 0.05 was used as a cut-off point for statistically significant relationships. All variables such as socio-demographic, behavioural risk factors, and awareness of strategies for HIV prevention programs that had p < 0.20 were included in the multivariable logistic regression analysis. For the multivariate analysis, we added variables one by one to produce the multivariate model [48]. IBM SPSS Statistics 25.0 was used to analyze the data.

### Ethical consideration

This study was approved by the Research Ethics Committee of Muhimbili University of Health and Allied Sciences (MUHAS) (Ref. No. DA.282/298/01.C/). Privacy and confidentiality of the respondents were ensured by collecting data in a private room and de-identifying the personal information from all study documents. We informed respondents about the voluntary nature of the study as well as the risks and benefits they might expect from their participation in the study. We also obtained written consent from each respondent.

## Results

### Socio-demographic characteristics of respondents

Two hundred sixty (260) PWIDs responded to the questionnaire. The mean age of the respondents and the social network size of the seeds was 39.0 years and 18.4 people with a standard deviation (SD) of ±7.5 and ±25.6 respectively. Most of the respondents were male (n = 232, 89.2%). The majority of the respondents had attained a primary level of education (n = 176, 67.7%) and (n = 86) 33.1% were separated from marital relationships. The majority of the respondents (n = 220, 84.6%) were self-employed. Among the self-employed, (n = 121) 55% were doing petty business such as picking and selling empty water bottles, ornaments, and shoe hawkers. In this study, (n = 92) 35.4% of respondents were from Ilala Municipality and more than half (n = 143, 55.0%) were living in family houses. Nearly (n = 155) 60% had a history of having been arrested by police officers for various criminal offences **(Table 1)**.

**Table 1:** Sociodemographic characteristics of the respondents (N = 260).

Variables	Frequency, N (%)
Age Mean age = 39.0, SD = ±7.5	
Gender	
Male	232 (89.2)
Female	28(10.8)
Educational level	
Never educated	26(10)
Primary	176 (67.7)
Secondary	55(21.2)
Above secondary	3(1.2)
Marital status	
Single	85(32.7)
Married	15(5.8)
Cohabiting	59(22.7)
Separated	86(33.1)
Divorced	4(1.5)
Widowed	11(4.2)
Activity for daily living	
Employed	6(2.3)
Self-employed	220(84.6)
Jobless	34(13.1)
Self-employment activities	
Petty business	121(55.0)
Technical	19(8.6)
Labourer	53(24.1)
Mini-bus conductors(Daladala)	18(8.2)
Sex work	9(4.1)
Residence	
Kinondoni	78(30.0)
Ilala	92(35.4)
Temeke	40(15.4)
Ubungo	46(17.7)
Kigamboni	4(1.5)
Housing status	
Family house	143(55.0)
Own house	7(2.7)
Rented house	73(28.1)
No permanent house	37(14.2)
Ever arrested by police	
Yes	155(59.6)
No	105(40.4)

### Injecting drugs, risky sexual behaviour, and practices

Almost half (n = 121, 46.5%) of the study sample injected drugs for more than ten years and (n = 130) 50% were injecting three times per day. Almost half (n = 133, 51.2%) of the respondents shared syringes or needles when using the drugs. Of those who shared syringes and needles, (n = 74) 55.6% shared more than three times during the period of drug use. About 5% (n = 14,) of the study sample shared blood in a syringe from a person who had just injected a drug to relieve addiction pains; a technique known as “flash blood”. Most respondents (n = 229, 88.1%) cleaned the syringe and needle using cold water before injecting the drugs. The most commonly injected drug was heroin (n = 228, 87.7%). In addition to injection drugs, about half (n = 128, 49.9%) of the study sample were using alcohol. A good number of respondents (n = 105, 40.5%) had one sexual partner in the past six months. However, half (n = 130, 50.2%) of them had practised sexual intercourse six months before the commencement of the study. Of those who had engaged in sexual intercourse, (n = 69) 53.1% did not use any kind of protection against HIV and (n = 43) 62.3% had participated in sexual intercourse without using a condom more than three times during the past six months. Only (n = 42) 16.2% of the respondents were involved in commercial sex work. However, most (73.8%) of the study sample reported being at high risk of HIV infection **(Table 2).**

**Table 2:** Injecting drugs, risky sexual behaviour, and practices (N = 260).

Variables	Frequency, N (%)
Duration of injecting drugs (Years)	
1–5	46(17.7)
6–10	93(35.8)
More than 10	121(46.5)
Frequency of injecting drugs per day	
One	1
Twice	15(5.8)
Three times	130(50.0)
More than three times	114(43.8)
Shared syringe or needle	
Yes	133 (51.2)
No	127 (48.8)
Frequency of sharing	
Once	9(6.8)
Two times	20(15.04)
Three times	30(22.6)
More than three times	74(55.6)
Withdrawing blood in a syringe(Flashblood)	
Yes	14(5.4)
No	246(94.6)
*Practiced Risky behaviour when injecting drugs	
Assisted by another person to inject	172(66.2)
Used unsafe syringe and needle	117(45.0)
Shared razor blade	136(52.3)
Used unclean needle and syringe	60(23.1)
Cleaned needle and syringe with cold water	229(88.1)
Never practiced	17(6.5)
*Types of drugs commonly injected	
Cocaine	59 (22.7)
Heroine	228 (87.7)
Mixture of heroin and cocaine	8(3.1)
Valium	5(1.9)
Others	5(1.9)
*Types of drugs used	
Cannabis	128(49.2)
Alcohols	128(49.9)
Khat	11(4.2)
Never	16(6.2)
Others	79(30.4)
Number of sexual partners in the past 6 months	
None	28(10.8)
One	105(40.5)
Two	52(20.1)
Three	34(13.1)
More than three	40(15.4)
Had sexual intercourse in the past 6 months	
Yes	130 (50.2)
No	129(49.8)
Used protection against HIV in the past 6 months	
Yes	61(46.9)
No	69 (53.1)
Frequency of condomless sexual intercourse in the last 6 months	
One	2(2.9)
Twice	1(1.4)
Three times	23(33.3)
More than three times	43(62.3)
Ever sold sex	
Yes	42(16.2)
No	218(83.8)

*Respondent chose more than one option.

### Awareness of the strategies for HIV prevention programs

More than half (n = 133, 51.2%) of the respondents were aware of the strategies for HIV prevention programs. Of those who were aware of the HIV prevention program, 63.9% (n = 85) were conversant with Tanzania AIDS Prevention Program (TAPP) followed by medication-assisted treatment(MAT) (n = 68, 51.1%). The majority (n = 213, 81.9%) of the respondent had tested for HIV in their lifetime. Of those who tested for HIV, 48.6% (n = 103) had tested in the past three months. More than 90% (n = 195) of the respondents reported being HIV-negative **(Table 3).**

**Table 3:** Awareness of strategies for prevention of HIV infection (N = 260).

Variables	Frequency, N (%)
Awareness of HIV prevention Programs for PWIDs	
Yes	133(51.2)
No	127(48.8)
*Awareness on	
**MAT	68 (51.1)
DIC	52(39.1)
TAPP	85(63.9)
NSEP	66(49.6)
Others	9(6.7)
Ever tested HIV in your lifetime	
Yes	213(81.9)
No	47(18.1)
Tested HIV in the last 3 months	
Yes	103(48.6)
No	110(51.4)
Aware of the HIV status	
Negative	195(91.5)
Positive	18(8.5)
Self-rating of risk for HIV infection	
Low risk	68 (26.2)
High risk	192 (73.8)

*Respondent had more than one options.

**MAT = Medication assisted treatment, DIC = Drop-In- centre, NSEP = Needle, and Syringe Exchange Program, TAPP = Tanzania AIDS Prevention Program.

### Awareness and willingness to use PrEP

Only 16.2% (n = 42) of the respondents had heard about (aware of) PrEP before the current study. Of those who heard about PrEP, most (n = 34, 82.9%) heard from friends/peers within 3 months (n = 20, 47.7%) before the present study. In addition, almost all (n = 40, 97.6%) of those who heard PrEP did not use it. The common reason for not using PrEP was a lack of awareness of where to get it (n = 23, 56.1%). On the other hand, most (n = 239, 91.9%) respondents were willing to use PrEP if would be available. A high proportion (n = 225, 94.1%) of respondents would use PrEP regardless of side effects and 76.1% (n = 194) would use PrEP together with condoms. The majority (n = 233, 97.5%) of the respondents would use PrEP with HIV testing every 3 months. About three-quarters (n = 186,71.4%) of the respondents said would be very easy for them to take pills every day and 79.6% (n = 207) would prefer to take injectable PrEP if it is made available. The common reasons reported by the respondent for choosing injectable PrEP were having experience with injection (27.1%), it taking a long interval between doses (n = 72, 34.8%), being more reliable than pills (n = 19, 9.2%), and being easy to use (n = 60, 29.0%) **(Table 4)**.

**Table 4:** Awareness and willingness to use PrEP among PWIDs (N = 260).

Variables	Frequency, N (%)
Ever heard about PrEP	
Yes	42(16.2)
No	218(83.8)
*Source of information about PrEP	
Media	6(14.6)
Friends/peers	34(82.9)
Health care providers	7(17.1)
The time when heard about PrEP	
Within three months ago	20(47.7)
3–6 month	13(31.0)
7–12 months	6(14.2)
For more than a year	3(7.1)
Used PrEP	
Yes	2(2.4)
No	40(97.6)
Reasons why did not use PrEP	
Did not know where to get it	23(56.1)
No enough information	12(29.3)
Do not believe in the PrEP	2(4.9)
Undecided	1((2.4)
Low risk	2(4.9)
Willing to use PrEP	
Yes	239(91.9)
No	21(8.1)
Willing to use PrEP regardless of the side effects	
Yes	225(94.1)
No	14(5.9)
Willing to take PrEP and use condoms	
Yes	194(81.2)
No	45(18.8)
Willilling to use PrEP and test for HIV every 3 months	
Yes	233(97.5)
No	6(2.5)
How easy to take pills every day	
Very easy	186(71.4)
Somewhat easy	23(8.9)
Somewhat difficulty	23(8.9)
Very difficulty	28(10.8)
Preference for pills or injection	
Pills	53(20.4)
Injection	207(79.6)
Reasons for the choice of injectable PrEP	
Experienced with injection	56(27.1)
Takes a long time	72(34.8)
More reliable than pills	19(9.2)
Easy to use	60(29.0)

*Respondent chose more than one option.

### Factors associated with awareness and willingness to use PrEP among the respondents

Awareness of PrEP was associated with gender(*X*^*2*^(1) = 9.40, *p* = .002), HIV prevention program (*X*^*2*^(1) = 11.65, *p* = < .001), selling sex(*X*^*2*^(1) = 6.68, *p* = .010) and frequency of condomless sexual intercourse(*X*^*2*^(1) = 9.00, *p* = .029). Age group, educational level, marital status, the risk level of HIV infection and number of sexual partners were not statistically associated with PrEP awareness

On the other hand, gender (*X*^*2*^(1) = 12.10, *p* = < .001), HIV prevention programs (*X*^*2*^(1) = 6.84, *p* = .006), risk level of HIV infection (*X*^*2*^(1) = 11.56, *p* = .001) and number of sexual partners (*X*^*2*^(4) = 14.26, *p* = .006) were statistically associated with willingness to use PrEP. Additionally, selling sex (*X*^*2*^(1) = 15.31, *p* = .021) and frequency of condomless sexual intercourse (*X*^*2*^(3) = 9.38, *p* = .025) showed statistical relationship with willingness to use the PrEP. After adjusting for confounding factors, only gender*(P* = 0.046) was related to awareness of PrEP and HIV prevention programs (*p* = 0.009), the risk level of HIV infection(*p* = < .001), number of sexual partners(*p* = 0.0459) and frequency of condomless sex(*p* = 0.032) for willingness to use PrEP. Other factors were not statistically significant **(Table 5).**

**Table 5:** Factors associated with awareness and willingness to use PrEP among the respondents.

Factors	Awareness about PrEP					Willingness to use PrEP				
	Yes, N (%)	No, N (%)	Chi-square (X^2^)	P value	Adjusted P value	Yes, N (%)	No, N (%)	Chi-square (X^2^)	P value	Adjusted p-value
Age group										
20–29	2(4.9)	27(12.3)	2.08	0.556		26(89.7)	3(10.3)	1.52	0.677	
30–39	15(36.6)	75(34.2)	3			81(90.0)	9(10.0)	3		
40–49	21(51.2)	99(45.2)				113(94.2)	7(5.8)			
50–59	3(7.3)	18(8.2)				19(90.5)	2(9.5)			
Gender										
Male	31 (75.6)	201(91.8)	9.40	0.002	.046	218(94.0)	14(6.0)	12.10	< .001	0.073
Female	10(24.4)	18(8.2)	1			21(75.0)	7(25.0)	1		
Education level										
Never	3(7.3)	23(10.5)	1.19	0.755		23(88.5)	3(11.5)			
Primary	30(73.2)	146(66.7)	3			165(91.5)	15(8.5)	1.24	0.743	
Secondary	8(19.5)	47(21.5)				52(94.5)	3(5.5)	3		
Above secondary	0(0.0)	3(1.4)				3(100.0)	0(0.)			
Marital status										
Single	9(22.0)	76(34.7)	5.94	.312		75(88.2)	10(11.8)	2.69		
Married	2(4.9)	13(5.9)	5			14(93.3)	1(6.7)	5	0.748	
Cohabiting	10(24.4)	49(22.4)				55(93.2)	4(6.8)			
Separated	16(39.0)	70(32.0)				81(94.2)	5(5.8)			
Divorced	2(4.9)	2(0.9)				4(100.0)	0(0.0)			
Widowed	2(4.9)	9(4.1)				10(90.9)	1(9.1)			
HIV prevention program										
Yes	31(75.6)	102(46.6)	11.65	.001	< .001	128(96.2)	5(3.8)	6.84	.009	.009
No	10(24.4)	117(53.4)	1			111(87.4)	16(12.6)	1		
The risk level of HIV infection										
Low risk	13(31.7)	28(68.3)	0.78	.378		56(82.4)	12(17.6)	11.56	.001	< .001
High risk	55(25.1)	164(74.9)	1			183(95.3)	9(4.7)	1		
Number of sexual partners										
None	3(7.3)	25(11.4)	5.46	.244		23(82.1)	5(17.9)			
One	13(31.7)	92(42.0)	4			97(92.4)	8(7.6)	14.29	.006	0.459
Two	13(31.7%)	40(18.3)				53(100.0)	0(0.0)	4		
Three	4(9.8)	30(13.7)				33(97.1)	1(2.9)			
More than three	8(19.5)	32(14.6)				33(82.5)	7(17.5)			
Selling sex										
Yes	12(29.3)	29(13.2)	6.68	.010	.427	34(82.9)	7(17.1)	5.31	.021	0.820
No	29(70.7)	190(86.8)	1			205(93.6)	14(6.4)	1		
Frequency of condomless sex										
Once	5(12.2)	17(7.8)	9.00	.029	0.150	22(100.0)	0(0.0)	9.38	0.025	0.032
Twice	3(7.3)	14(6.4)	3			15(88.2)	2(11.8)	3		
Three times	20(48.8)	64(29.2)				82(97.6)	2(2.4)			
More than three times	13(31.7)	124(56.6)				120(87.6)	17(12.4)			

## Discussion

To our knowledge, this is the first study of PrEP awareness and willingness among Tanzanian PWIDs, a marginalized and key population at high risk for HIV. In the recruited sample of PWIDs, we found a relatively low awareness of PrEP, and a very high willingness to use this prevention strategy. Awareness and willingness to use PrEP were associated with gender, awareness of HIV prevention programs, the risk level of HIV infection, number of sexual partners, selling sex, and frequency of condomless sex. The majority of PWIDs preferred long-acting injectable PrEP because of their experience in injecting practice.

The relatively low awareness of PrEP among the study sample may be due to inadequate formal or planned outreach activities for PWIDs to access health services in the community. This can be described by the lack of integration of HIV PrEP services in harm reduction programs such as medication-assisted treatment (MAT) and needle and syringe exchange program (NSEP) which are common access points for PWIDs [49,50]. Likewise, studies among female barmaids and adolescent girls, and young women (high-risk populations) in Tanzania revealed low awareness of PrEP [34,51]. This implies that a sensitization campaign on awareness and utilization of PrEP in the target high-risk population in Tanzania is still at a rudimentary stage. Similarly, low awareness (6.1%) of PrEP among PWID was reported in India [52] and Philadelphia (12.4%) [53]. In contrast to our study results, moderately high awareness of PrEP among PWID has been reported in New York City(31%) [50] and Los Angeles(40%) [54], both from United State. The moderately high awareness of the PrEP in the USA can be described by the fact that the PrEP sensitization and implementation campaign among PWID started earlier [55,56] than in most African countries [57]. This implies that social networks (specifically NSEP and MAT) are needed as a means for effective spreading messages about prevention materials such as PrEP as reported in a study by Walter et al [58] in the United States of America.

As has been reported in our study, gender, awareness of HIV prevention programs, selling sex, and frequency of condomless sex are associated with awareness of PrEP among PWIDs. This can be described by the fact that our study sample considered themselves a high-risk population. In Philadelphia (US), awareness of PrEP among PWID has been reported to be associated with factors such as education level, sharing paraphernalia, STI treatment, and drug treatment [53]. The mismatch of these factors may be described by the differences in socio-cultural context between the two settings. However, the predictive factors in our study are important for planning educational materials for improving awareness of PrEP among the study population.

Despite low awareness, a majority of PWIDs expressed willingness to use oral PrEP. Similarly, a higher willingness to use PrEP among PWIDs has been reported in other countries [52,54,59]. Most PWIDs in our study population are interested to use PrEP to prevent HIV infection. While Bellundi et al [52] reported sharing needles and hazardous alcohol use was associated with increased willingness to use PrEP, our study reported selling sex, frequency of condomless sex, and having more than one sexual partner are associated with increased interest in PrEP, suggesting that PWID have insight into their own HIV risk. The results also make us reflect on the potential role of PrEP within a comprehensive package of HIV prevention, treatment, and care services. Specifically, if future PrEP programs can enrol PWIDs with increased vulnerability to HIV infection, then such programs may be successful in deterring incident cases in the larger PWID community. While Walters et al [50], suggested social networks such as NSEP as efficient means for disseminating messages about HIV prevention materials such as PrEP, more studies are required to explore the avenue for PrEP dissemination in Tanzania. Therefore, these results provide only preliminary information awareness and willingness to use PrEP among PWIDs in Tanzania.

Although 71.4% of our study sample would easily take oral PrEP every day to prevent HIV infection, a slightly high proportion (79.6%) prefer to take injectable medication. The injectable formulation is not yet introduced in low-income countries, particularly in Tanzania [60]. However, demonstration studies in other countries have shown a significant preference for long-acting injectables over oral PrEP among transgender, men who have sex with men, and youth [20,61–63] but few have been reported in PWIDs [52,64]. Our results showed a high proportion of preference for long-acting PrEP compared to that reported in India(31%) [52]. The reason for this difference between our results and that in India needs further investigation. The most commonly cited reasons for the choice of injectable PrEP in our study sample include experience with injection, the long interval between the next dose, more reliability of injection, and ease to use. Our results indicate that long-acting injectable PrEP is the most likely formulation to be used by PWIDs in Tanzania if it will be available.

Concerning the awareness of programs for the prevention of HIV infection, the majority of our study sample were aware of the Tanzania AIDS Prevention Program (TAPP), a program that started in 2007 to implement prevention and outreach activities among PWID in Dar es Salaam [5]. While half of PWIDs are conversant with medication-assisted treatment (MAT clinic) which started operating in 2011 [65], integrated methadone and antiretroviral therapy (IMAT) strategy has shown an effective HIV care model for PWID in Tanzania [66]. Our study results suggest that the effective provision of PrEP among PWIDs can be easily accessible through the IMAT program. Regarding the high (73.8%) self-rating of risk for HIV infection in our study, we are convinced that combination interventions including sexual risk reduction and harm reduction as proposed by Fraser et al [67] are needed to reduce /eliminate HIV among PWID in Tanzania.

The current study has multiple implications for HIV prevention policy and research. First, given the relationship between the number of risk factors and willingness to use PrEP, it is imperative to devise guidelines that focus on the integration of PrEP with other health services. Integrating PrEP services across the healthcare systems may lead to improved PrEP awareness and utilization among PWID as reported in other studies [68–70]. Second, MAT clinics are good avenues for providing information and education related to HIV prevention among PWIDs [71,72]. To increase the efficiency of HIV prevention among PWIDs, provision of education to increase awareness and uptake of PrEP should be introduced particularly in low-resource settings countries such as Tanzania. Lastly, while this paper only focuses on oral PrEP, future research should also assess the acceptability of injectable PrEP for PWID, as their acquaintance with injection may make the formulation more practical.

The present study’s results must be construed in light of the following limitations. Specifically, the data presented were collected with a cross-sectional approach; therefore, any conclusions based on causality or directions of specific relationships cannot be made. Although one of the benefits of RDS is that it is likely to reduce bias by making extensive recruitment chains that link relevant groups, oversampling may have occurred from well-connected social networks that may be different from the target population, particularly to geographical location, income, socioeconomic status, and gender. Also, the data is unweighted because assumptions needed for weighting were not met, and therefore is not a probability sample [73–75] making generalizability questionable. However, the results from this study have provided insights into the need for PrEP for the PWID population in Tanzania

### Conclusion

Despite the low awareness of PrEP among PWIDs, the high willingness for PrEP use is a promising step toward HIV prevention infection. PrEP provision to PWIDs in Tanzania is feasible considering that effective dissemination of information to the target population is implemented. Future research should assess the acceptability of injectable PrEP for PWID, as their acquaintance with injection may make the formulation more practical

## Supporting information

S1 File Eligibility screening form for people who inject drugs. (DOCX)

S2 File Data collection tool. (DOCX)
