# Supplementary material for: Awareness and willingness to use HIV oral pre-exposure prophylaxis among people who inject drugs in Dar es Salaam, Tanzania: A cross-sectional survey
Source: PLOS Glob Public Health. 2022 Nov 22;2(11):e0000776. doi: 10.1371/journal.pgph.0000776 (PMC10121179; doi:10.1371/journal.pgph.0000776)
Supplement: S1 File — (DOCX) [file pgph.0000776.s001.docx]

Eligibility Screening Form – PWID

**Participants Code Number _ _ _ _ _ _ _ _ _ _ _ _ _ _ _ _ _ _ _ _ _ _ _ _ _ _**

| **Name of the interviewer** | | **Date of Interview** | | **Place:_______________** | |
| --- | --- | --- | --- | --- | --- |
| 1 | He/she has been involved in this research  Yes / No (Circle one) | | Deserves | | Does not deserve |
| 2 | He/she has valid coupons  *Coupon expiration date:*  _ _ / _ _/ **2021** | | Deserves | | Does not deserve |
| 3 | He/she is 18 years or older  (*How old are you?)*________ | | Deserves | | Does not deserve |
| 4 | He/she injected himself with drugs six months ago  (*When was the last time you injected yourself with drugs?)* | | Deserves | | Does not deserve |
| 5 | He/she lives in Dar es Salaam  *(What area does he live in?)* | | Deserves | | Does not deserve |
| 6 | He has lived in Dar es Salaam for the past six months  *(How long have you lived in Dar es Salaam?)* | | Deserves | | Does not deserve |
| IF ELIGIBLE CONDUCT CONSENT NOW | | | | | |
