# Supplementary material for: Awareness and willingness to use HIV oral pre-exposure prophylaxis among people who inject drugs in Dar es Salaam, Tanzania: A cross-sectional survey
Source: PLOS Glob Public Health. 2022 Nov 22;2(11):e0000776. doi: 10.1371/journal.pgph.0000776 (PMC10121179; doi:10.1371/journal.pgph.0000776)
Supplement: S2 File — (DOCX) [file pgph.0000776.s002.docx]

**A QUESTIONNAIRE FOR AWARNESS AND WILLINGNESS TO USE PrEP AMONG PEOPLE WHO INJECT DRUGS**

|  |  |  |  |  |
| --- | --- | --- | --- | --- |

**Section I: Sociodemographic information** (**Please put a TICK in the appropriate box)**

1. What is your age? …………………………..
2. Gender ……………………………
3. What is your level of education?

- Never gone to school
- Primary
- Secondary
- Above secondary

1. What is your marital status?

- Single
- Married
- Cohabiting
- Separated
- Divorced
- Widow/widower

1. What do you do for a living?

- I am employed
- I am self-employed
- I do not have a job

1. If you are self-employed, what activities do you do?
2. ________________________________________________________________________
3. ________________________________________________________________________
4. ________________________________________________________________________
5. Where do you live?

- Kinondoni
- Ilala
- Temeke
- Ubungo
- Kigamboni

1. What is the residence status?

- I live in a family house
- I live in my house (built)
- I have rented room/house
- I have no official residence

1. Have you been arrested by the police for any criminal activity in the last 6 months??

- Yes
- No

**Section II: Injecting drug behaviours and practice**

The next questions are related to the practice of injecting drugs (**Please put a TICK in the appropriate box**)

1. How long have you been injecting drugs?

………….years

1. How many times per day do you inject drugs?

……………times per day

1. Have you ever injected drugs using needles and/or syringes that had been previously used by someone else?

- Yes
- No

1. If yes, how often did you inject drugs using a needle or syringe used by someone else?

- Once
- Twice
- Three times

More than three times


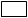


1. Have you ever practiced withdrawing blood in a syringe after injected and giving it to someone else in the past 6 months?

- Yes

No


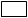


1. Which of the following practices have you used in your last injection? (SELECT ALL THAT APPLY)

- Helped by someone to inject
- Used unsterile needle and syringe
- Shared razor blade
- Injected with a syringe not cleaned
- Injected with a syringe that was first cleaned with water

1. Which types of injection drugs do you use? (SELECT ALL THAT APPLY)

- Cocaine
- Heroin
- Methamphetamine
- Speedball (Cocaine + Heroine)
- Tranquilizers (Valium)

Others (Please specify)


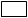


1. What other drug have you ever used in the past 6 months (SELECT ALL THAT APPLY)

- Marijuana
- Alcohol
- Khat
- Not applicable

Others (Specify)


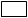


1. ________________________________________________________________________
2. ________________________________________________________________________
3. ________________________________________________________________________

**Section III: Awareness and strategies for prevention of HIV infection**

1. Are you aware of any HIV and AIDS prevention programs aimed at drug users?

- Yes

No


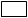


1. If yes in above question, which program are you aware of?

- Medication assisted treatment (MAT)
- Drop in Centre (DIC)
- Tanzania AIDS Prevention Program (TAPP)
- Needle and Syringe Exchange Program (NSEP)

Others (specify)


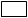


1. ________________________________________________________________________
2. ________________________________________________________________________
3. ________________________________________________________________________
4. Have you ever tested for HIV in your life time?

- Yes
- No

1. Have you tested for HIV in the last three months?

- Yes
- No

1. If yes, what were the test results?

- I am HIV negative
- I am HIV positive
- I did not pick up the results / I do not remember

1. Looking back on how you inject yourself, how much do you think you are at risk of contracting HIV?

- No risk
- Low risk
- Moderate risk
- High risk

**Section IV: Sexual Behaviour and practice**

1. How many sexual partners have you had in the past 6 months**?**

- None
- One
- Two
- Three

More than three


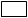


1. Have you had sexual intercourse in the past 6 months with someone else other than your regular sexual partner?

- Yes
- No

1. If yes in the question above, did you use any kind of protection??

- Yes
- No

1. If yes, what kind of protection did you use?

- Condoms
- PrEP
- Lubricants
- Saliva

1. How often have you had sexual intercourse without using any protection in the past 6 months?

- Once
- Two times
- Three times
- More than three times

1. Have you ever sold sex?

- Yes
- No

**Section IV: Awareness, willingness and acceptability to use PrEP**

1. Have you heard of PrEP for prevention of HIV?

- Yes
- No

1. If Yes, where did you hear it?


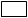
In the media


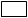
From friends /other people


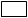
From health care providers


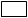
Others (specify)

1. ________________________________________________________________________
2. ________________________________________________________________________
3. ________________________________________________________________________
4. If Yes, how long ago did you hear about PrEP?

- Within the last 3 months
- 3-6 months ago
- 6-12 months ago
- Over a year ago
- More than two years ago

1. Have you ever used PrEP to reduce the risk of HIV infection?

- Yes
- No

1. If the answer is yes, when was the last time you used it?

___________________________________________________________________________

1. If the answer is no, what are some reasons why you did not use PrEP?
2. _______________________________________________________________________
3. ________________________________________________________________________
4. ________________________________________________________________________
5. ________________________________________________________________________

**If you have never heard of it before, here is a brief summary of PrEP:**

| Pre-exposure Prophylaxis (PrEP) is a method of HIV prevention that involves people who are not infected with HIV taking one pill every day. PrEP is already in use in several countries. This method of HIV prevention involves people who are not infected but who are at high risk, such as many people who inject themselves with drugs. PrEP users take this treatment in anticipation of HIV infection to reduce the risk of infection. |
| --- |

1. If PrEP was available in Tanzania for the prevention of HIV infection, would you intend to use it as a HIV prevention method?

- Yes

No


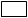


1. Would you take PrEP if it caused mild temporary side effects, such as headache, nausea, etc?

- Yes

No


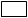


1. Would you take PrEP if you still had to use condoms to be fully protected from HIV and other STIs?

- Yes

No


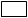


1. Would you take PrEP if it meant you would have to be tested for HIV every three months?

- Yes
- No

1. How easy would it be for you to take a pill each day, to prevent HIV infection?

- Very easy
- Somewhat easy
- Somewhat difficulty
- Very difficulty

1. If there is difficulty, what causes it?
2. ________________________________________________________________________
3. ________________________________________________________________________
4. ________________________________________________________________________
5. ________________________________________________________________________

1. If you had a choice to use a daily pill or an injection every one to two months to protect yourself from HIV, which one would you choose?

- Prefer the pill
- Prefer the injection

1. If you would prefer to use **daily pills** to protect yourself from HIV, what are the reasons for your choice? (PLEASE WRITE YOUR ANSWERS IN THE SPACE PROVIDED)
2. ________________________________________________________________________
3. ________________________________________________________________________
4. ________________________________________________________________________
5. ________________________________________________________________________
6. ________________________________________________________________________
7. If you would prefer to use **an injection every one to two months** to protect yourself from HIV, what are the reasons for your choice? (PLEASE WRITE YOUR ANSWERS IN THE SPACE PROVIDED)
8. ________________________________________________________________________
9. ________________________________________________________________________
10. ________________________________________________________________________
11. ________________________________________________________________________
12. ________________________________________________________________________

**Personal Network Size (Degree)**

1. Number of people the respondent knows within the target population……………………….

**Respondent's Serial Number**

1. Serial number of the coupon the respondent was recruited with……………………………..

**Respondent's Recruiting Serial Numbers**

1. Serial numbers from the coupons the respondent is given to recruit others…………………..

**Thank you for participation**
